# Spontaneous nystagmus with an upbeat component: Central or peripheral vestibular disorders?

**DOI:** 10.3389/fneur.2023.1106084

**Published:** 2023-02-23

**Authors:** Xia Ling, Yue-Xia Wu, Yu-Fei Feng, Tong-Tong Zhao, Gui-Ping Zhao, Ji-Soo Kim, Xu Yang, Zhao-Xia Wang

**Affiliations:** ^1^Department of Neurology, Peking University First Hospital, Beijing, China; ^2^Department of Neurology, Aerospace Center Hospital, Peking University Aerospace School of Clinical Medicine, Beijing, China; ^3^Research Administration Team, Seoul National University Bundang Hospital, Seongnam, Republic of Korea; ^4^Dizziness Center, Seoul National University Bundang Hospital, Seongnam, Republic of Korea

**Keywords:** vestibular disorders, topical diagnosis, etiology, upbeat nystagmus, pure upbeat nystagmus, SN with predominant upbeat component

## Abstract

**Objective:**

To determine the topical diagnosis and etiologies of spontaneous nystagmus (SN) with an upbeat component.

**Methods:**

We retrospectively recruited 43 patients with SN with an upbeat component at a university hospital in China from 2020 to 2022. SN with an upbeat component was divided into pure upbeat nystagmus (UBN), SN with a predominant upbeat component, and SN with a non-predominant upbeat component. We analyzed their clinical and neurotologic findings and the final diagnosis.

**Results:**

Fourteen (32.6%) of them showed pure UBN, while 29 (67.4%) exhibited SN mixed with an upbeat component, mixed upbeat-horizontal in 15, mixed upbeat-horizontal-torsional in 13, and upbeat-torsional in the remaining one. Pure UBN and SN with a predominant upbeat component were more common in central than in peripheral vestibular disorders [16 (80.0%) vs. 0 (0%), Chi-Square test, *p* < 0.001]. Central vestibular disorders were diagnosed in 20 (46.5%) patients, peripheral in 14 (32.6%), and undetermined in nine (20.9%) patients. The underlying causes mainly included acute unilateral peripheral vestibulopathy (*n* = 11), posterior circulation infarction (*n* = 9), benign recurrent vertigo (*n* = 4), vestibular migraine (VM, *n* = 3), and VM of childhood (*n* = 2).

**Conclusion:**

SN with an upbeat component can be seen in both central and peripheral vestibular disorders. Pure UBN was a characteristic sign of central vestibular dysfunction. Central vestibular disorders should be highly suspected when patients show pure UBN or SN with a predominant upbeat component.

## Introduction

Vertigo and dizziness are the most common symptoms of vestibular dysfunction, while one of the most frequent signs is spontaneous nystagmus (SN). Evaluation of SN is an important indicator in differentiating central and peripheral vestibular disorders (1). SN can be classified into pure (horizontal, vertical, or torsional) and mixed nystagmus according to the component of nystagmus. SN with an upbeat component could be divided into pure UBN, SN with a predominant upbeat component, and SN with a non-predominant upbeat component.

Pure UBN and SN with a predominant upbeat component have been reported in patients with brainstem infarction, brainstem hemorrhage, Wernicke's encephalopathy, multiple sclerosis, tumors, episodic ataxia type 2, cerebellar degeneration, Creutzfeldt-Jakob disease, brainstem encephalitis, epilepsy, Behcet's syndrome, Chiari malformation, meningitis, congenital, vestibular migraine (VM), organophosphate toxicity, tobacco, drugs, canalith jam involving the anterior semicircular canal, Meniere's disease (MD), and delayed endolymphatic hydrops (2–11). SN with a non-predominant upbeat component has been reported in patients with both central and peripheral lesions, including lateral medullary infarction and medial medullary infarction (12, 13), and superior vestibular neuritis (VN) (14–16). Few studies have analyzed the topical diagnosis and etiologies of all kinds of SN with an upbeat component and the differences between nystagmus types in peripheral and central vestibular disorders (5–7, 17–20). Hence, we retrospectively analyzed the medical history and examination findings in 43 patients with SN with an upbeat component and explored the topical diagnosis, possible etiologies, and mechanism of SN with an upbeat component.

## Patients and methods

### Patient selection

We retrospectively reviewed the etiology database of dizziness patients established in our hospital for patients with SN with an upbeat component who either visited the emergency department or the outpatient clinic of the Department of Neurology in our hospital from July 2020 to August 2022. Fifty-three consecutive patients were identified in this time period. Ten patients had to be excluded because of strong horizontal nystagmus but no apparent UBN when performing the video-oculography (VOG) test (*n* = 4), only described UBN on bedside examination but no apparent UBN when performing the VOG test (*n* = 4), lack of essential laboratory tests (*n* = 2). Eventually, 43 patients with SN with an upbeat component were included.

### Clinical data collection

The medical records of 43 patients with SN with an upbeat component were reviewed, including disease course, onset form, duration, frequency of attacks, precipitating/relieving factors, symptoms, signs, history, eye movement examination, caloric test, video head impulse test (vHIT, Interacoustics, Middelfart, Denmark), vestibular-evoked myogenic potentials (VEMPs), head magnetic resonance imaging (MRI), three-dimensional fluid-attenuated inversion recovery magnetic resonance imaging (3D-FLAIR MRI) and serum immunology test results and diagnosis. 2D-VOG (Interacoustics, Middelfart, Denmark) was performed for the detection of SN, gaze-evoked nystagmus, saccades, smooth pursuit, optokinetic, head-shaking nystagmus, and positional test. All patients underwent VOG examination during the acute phase or attack phase. The VOG examination was performed on the day of the visit for patients with acute attacks if they could cooperate with the examination. For patients with severe dizziness/vertigo that cannot cooperate with the examination, a VOG test should be performed within seven days of the latest attack.

All experiments followed the tenets of the Declaration of Helsinki and were approved by the Institutional Review Board of Aerospace Center Hospital.

### Classification of SN with an upbeat component

SN with an upbeat component could be divided into pure UBN, SN with a predominant upbeat component, and SN with a non-predominant upbeat component. Since we could not quantify the torsional component of nystagmus due to using a 2D video-oculography, the torsional component was determined by observing the video clips by two experiment neurologists. We mainly focused on comparing the intensity of horizontal and vertical components. When there was only an upbeat component, we defined it as pure UBN. When the intensity of the upbeat component of mixed SN was greater than that of the horizontal component, we defined it as SN with a predominant upbeat component. When the intensity of the upbeat component of mixed SN was equal to or lesser than that of the horizontal component, we defined it as SN with a non-predominant upbeat component.

### Diagnosis

According to the time characteristics of vestibular symptoms onset, patients can be classified into acute, episodic, or chronic vestibular syndrome (AVS, EVS, or CVS) (21). All diagnoses were made by the senior authors (ZXW and XY) according to widely accepted diagnostic criteria for each vestibular disorder or the international classification of vestibular disorders (ICVD) criteria when available (22–26). The published diagnostic criteria consensus includes acute unilateral vestibulopathy (AUVP)/VN (26), persistent postural-perceptual dizziness (PPPD) (23), VM (25), VM of childhood (24), and MD (22). Besides, probably labyrinthine infarction was diagnosed in older patients with sudden onset of unilateral deafness and vertigo, especially when there is a history of stroke or known vascular risk factors (27). Benign recurrent vertigo (BRV) was diagnosed when patients showed spontaneous rotational vertigo or instability; symptoms that were not triggered by changes in position, lasting longer than 1 min; normal audiogram or symmetric hearing loss; no cochlear symptoms (tinnitus or stuffiness) during the attack phase; no migraine or migraine aura in the acute phase (26, 28). Isolated acute unilateral utricular vestibulopathy was diagnosed in patients with acute onset of postural imbalance, which can be diagnosed by ocular VEMP (26).

### Statistical analyses

All the statistical analyses were performed using SPSS software (version 20.0, IBM SPSS Statistics, N.Y., USA). Continuous variables were expressed as a mean ± SD for parametric values or a median (range) for non-parametric ones. Counting variables were expressed as a percentage. The normality of the data was determined using the Shapiro-Wilk test. The Chi-square test was adopted to compare the proportion of the SN with a predominant upbeat component between peripheral and central disorders. *p* < 0.05 was considered significant.

## Results

Forty-three patients showing SN with an upbeat component were included in the study. There were 26 (60.5%) males and 17 (39.5%) females, with a mean age of 47.0 ± 16.5 years (range 12–78).

### Peripheral and central vestibular disorders

Central vestibular disorders were diagnosed in 20 (46.5%) patients, peripheral in 14 (32.6%), and undetermined in 9 (20.9%) patients. Among 14 patients with peripheral vestibular disorders, the underlying causes included AUVP (*n* = 11, superior AUVP in 10 and complete AUVP in one), probable labyrinthine infarction (*n* = 1), isolated acute unilateral utricular vestibulopathy (*n* = 1), and probable MD (*n* = 1).

Among 20 patients with central vestibular disorders, the underlying causes included posterior circulation infarction (PCI, *n* = 9), VM (*n* = 3), VM of childhood (*n* = 2), Wernicke's encephalopathy (*n* = 1), brainstem encephalitis (*n* = 1), postoperative fourth ventricle ependymoma (*n* = 1), autoimmune encephalitis (*n* = 1), transient ischemic attack (TIA; *n* = 1), and combination of VM and PPPD (*n* = 1). Among nine patients with undetermined central and /or peripheral vestibular disorders, the underlying causes included BRV (*n* = 4) and undetermined (*n* = 5).

**Figure 1:**
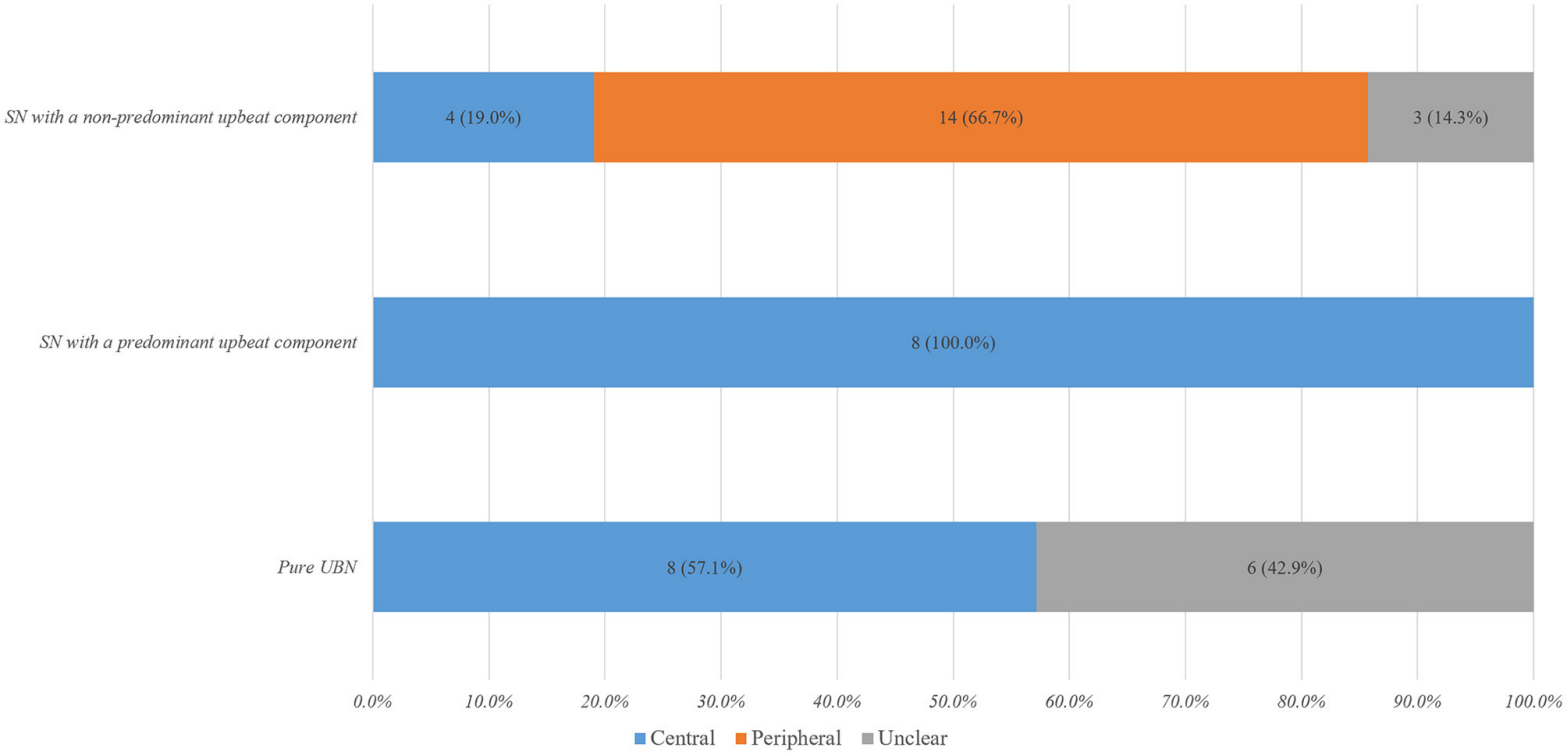
Characteristic distribution of spontaneous nystagmus with an upbeat component in vestibular disorders. SN, spontaneous nystagmus; UBN, upbeat nystagmus.

Fourteen (32.6%) of them showed pure UBN, while 29 (67.4%) exhibited SN mixed with an upbeat component, mixed upbeat-horizontal (UBN-HBN) in 15, mixed upbeat-horizontal-torsional (UBN-HBN-TN) in 13, and upbeat-torsional (UBN-TN) in the remaining one (Table 1). Among patients with pure UBN, central vestibular disorders were noted in eight (57.1%) and undetermined in six (42.9%). Patients with SN with a predominant upbeat component (the intensity of the upbeat component was greater than that of the horizontal component) were only in central vestibular disorders (8, 100%). Among patients with SN with a non-predominant upbeat component, peripheral vestibular disorders were noted in 14 (66.7%) patients, central in four (19.0%), and undetermined in three (14.3%) patients (Table 1, Figure 1).

**Table 1:** Etiology of patients with pure upbeat nystagmus and mixed nystagmus with an upbeat component.

**Nystagmus**	**Topical diagnosis**	**Etiological diagnosis**
Pure UBN (14, 32.6%)	Central (8, 57.1%)	Vestibular migraine (*n* = 2)
		Vestibular migraine of childhood (*n* = 2)
		Wernicke's encephalopathy (*n* = 1)
		Transient ischemic attack (*n* = 1)
		Autoimmune encephalitis (*n* = 1)
		Fourth ventricle ependymoma after surgery (*n* = 1)
	Unclear (6, 42.9%)	Benign recurrent vertigo (*n* = 2)
		Unclear (*n* = 4)
SN with a predominant upbeat component (8, 18.6%)	Central (8, 100%)	Mixed UBN, HBN, and TN (*n* = 1)
		Medial midbrain and pontine infarction (*n* = 1)
		Mixed UBN and HBN (*n* = 6)
		Lateral medullary infarction (*n* = 1)
		Lateral medullary and cerebellar infarction (*n* = 1)
		Ventral pontine infarction (*n* = 1)
		Brainstem encephalitis (*n* = 1)
		Vestibular migraine (*n* = 1)
		Vestibular migraine, PPPD (*n* = 1)
		Mixed UBN and TN (*n* = 1)
		Medial medullary infarction (*n* = 1)
SN with a non-predominant upbeat component (21, 48.8%)	Central (4, 19.0%)	Mixed UBN, HBN, and TN (*n* = 3)
		Medial medullary infarction (*n* = 1)
		Pontine tegmentum infarction (*n* = 1)
		Lateral medullary and cerebellar infarction (*n* = 1)
		Mixed UBN and HBN (*n* = 1)
		Medial medullary infarction (*n* = 1)
	Peripheral (14, 66.7%)	Mixed UBN, HBN, and TN (*n* = 8)
		Acute unilateral vestibulopathy (*n* = 8)
		Mixed UBN and HBN (*n* = 6)
		Acute unilateral vestibulopathy (*n* = 3)
		Probably labyrinthine infarction (*n* = 1)
		Isolated acute unilateral utricular vestibulopathy (*n* = 1)
		Probable Ménière's disease (*n* = 1)
	Unclear (3, 14.3%)	Mixed UBN, HBN, and TN (*n* = 1)
		Benign recurrent vertigo (*n* = 1)
		Mixed UBN and HBN (*n* = 2)
		Benign recurrent vertigo (*n* = 1)
		Unclear (*n* = 1)

HBN, horizontal nystagmus; PPPD, persistent postural-perceptual dizziness, SN, spontaneous nystagmus; TN, torsional nystagmus; UBN, upbeat nystagmus.

The horizontal component of SN was greater than the upbeat component in one patient with medial medullary infarction, and one patient with lateral medullary infarction, one patient with pontine tegmentum infarction, and one patient with lateral medullary and cerebellar infarction.

**Figure 2:**
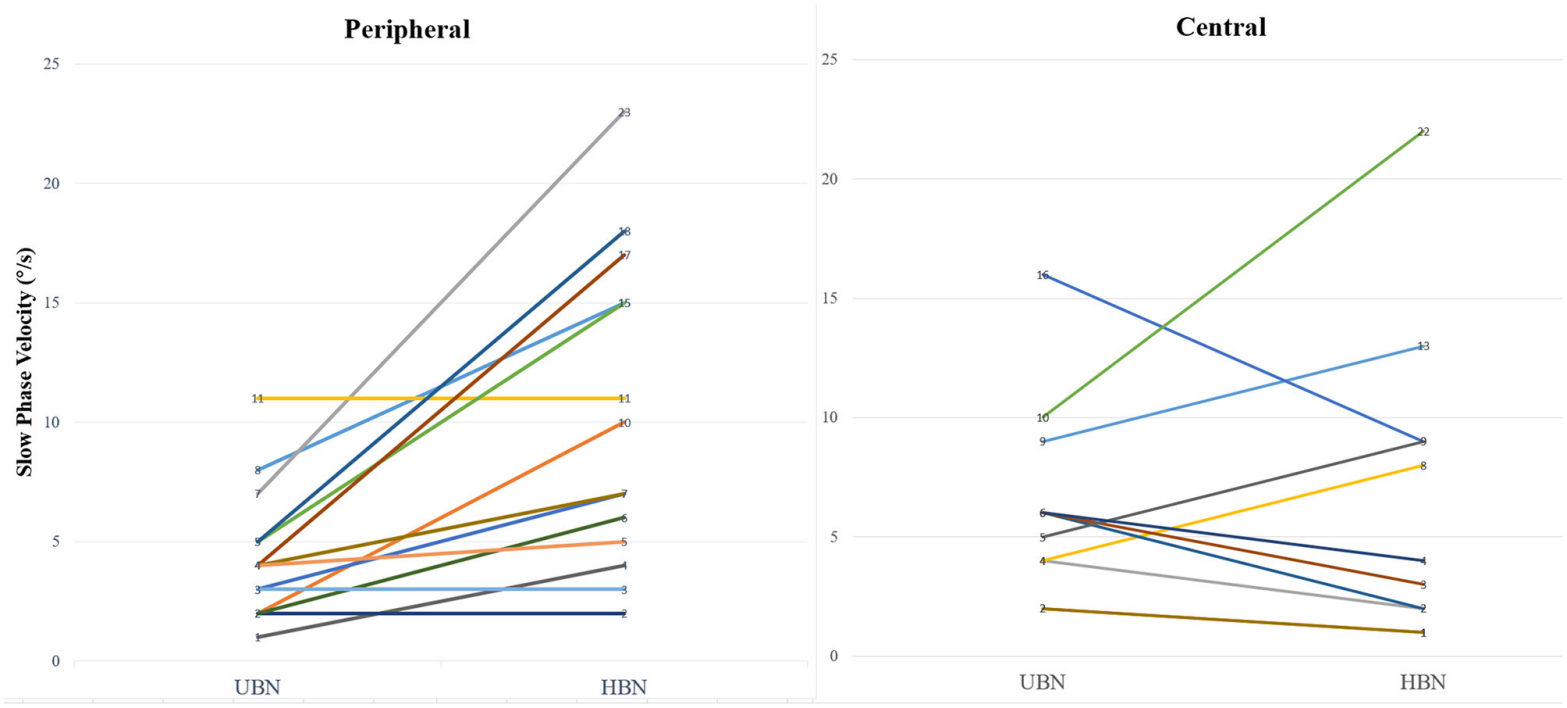
Comparison of the intensity of the horizontal and upbeat components of mixed spontaneous nystagmus in central and peripheral vestibular disorders. UBN, upbeat nystagmus; HBN, horizontal nystagmus.

Pure UBN and SN with a predominant upbeat component were more common in central than in peripheral vestibular disorders [16 (80.0%) vs. 0 (0%), Chi-Square test, *p* < 0.001]. The intensity of upbeat component was significantly weaker than that of the horizontal component in patients with peripheral mixed SN [4.4 ± 2.7°/s (median 4, range 1–11°/s) vs. 10.2 ± 6.4 °/s (median 8.5, range 2–23°/s), Wilcoxon Signed Ranks Test, *Z* = −2.928, *p* = 0.003]. There was no significant difference in the intensity of the upbeat component and horizontal component in patients with central mixed SN [6.4 ± 4.1°/s (median: 6, range: 2–16°/s) vs. 6.7 ± 6.5°/s (median: 4.0, range: 1–22), Wilcoxon Signed Ranks Test, *Z* = −0.045, *p* = 0.964, Figure 2].

### Vestibular syndromes

**Figure 3:**
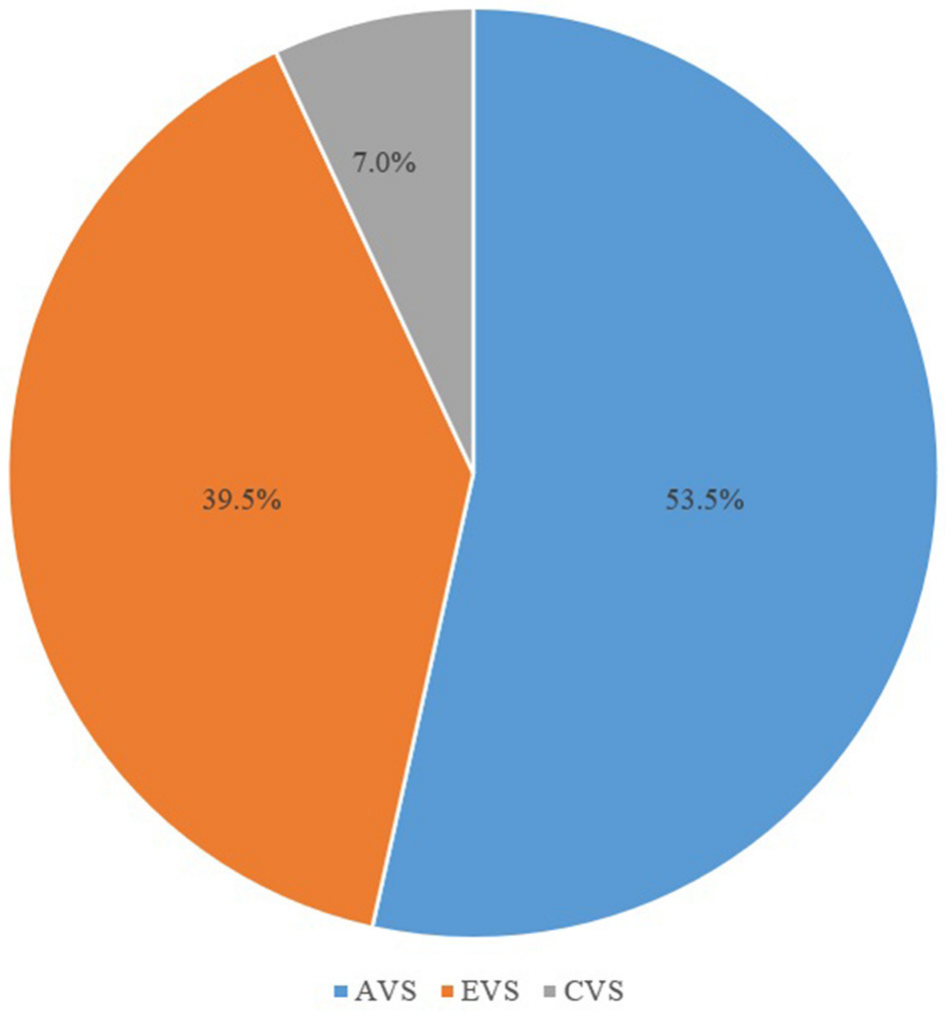
Proportion of vestibular syndrome types in patients showing spontaneous nystagmus with an upbeat component. AVS, acute vestibular syndrome; EVS, episodic vestibular syndrome; CVS, chronic vestibular syndrome.

According to the characteristics of the dizziness/vertigo attacks, 23 (53.5%), 17 (39.5%), and 3 (7.0%) of the patients with SN with an upbeat component had AVS, EVS, and CVS, respectively (Figure 3).

The AVS group included 11 patients with AUVP, nine patients with PCI (including medullary infarction in 4, pontine infarction in 2, medullary and cerebellar infarction in 2, and midbrain and pontine infarction in 1), one patient with probable labyrinthine infarction, one patient with isolated acute unilateral utricular vestibulopathy, and one patient with brainstem encephalitis.

The EVS group included BRV (*n* = 4), VM (n = 3), VM of childhood (*n* = 2), probable MD (*n* = 1), postoperative fourth ventricle ependymoma (*n* = 1), autoimmune encephalitis (*n* = 1), TIA (*n* = 1), and unclear causes (*n* = 4). The CVS group included Wernicke's encephalopathy (*n* = 1), concomitant VM and PPPD (*n* = 1), and unclear causes (*n* = 1; Table 2).

**Table 2:** Etiological distribution of patients with spontaneous nystagmus with an upbeat component.

**Vestibular syndrome**	**Topical diagnosis**	**Etiological diagnosis**
AVS (*n* = 23, 53.5%)	Peripheral (13, 56.5%)	Acute unilateral vestibulopathy (*n* = 11)
		Probably labyrinthine infarction (*n* = 1)
		Isolated acute unilateral utricular vestibulopathy (*n* = 1)
	Central (10, 43.5%)	Posterior circulation infarction (*n* = 9)
		Medullary infarction (*n* = 4)
		Pontine infarction (*n* = 2)
		Midbrain and pontine infarction (*n* = 1)
		Medullary and cerebellar infarction (*n* = 2)
		Brainstem encephalitis (*n* = 1)
EVS (*n* = 17, 39.5%)	Peripheral (1, 5.8%)	Probable Ménière's disease (*n* = 1)
	Central (8, 47.1%)	Vestibular migraine (*n* = 3)
		Vestibular migraine of childhood (*n* = 2)
		Postoperative fourth ventricle ependymoma (*n* = 1)
		Autoimmune encephalitis (*n* = 1)
		Transient ischemic attack (*n* = 1)
	Unclear (8, 47.1%)	Benign recurrent vertigo (*n* = 4)
		Unclear (*n* = 4)
CVS (*n* = 3, 7.0%)	Central (2, 66.7%)	Wernicke's encephalopathy (*n* = 1)
		Vestibular migraine, PPPD (*n* = 1)
	Unclear (1, 33.3%)	Unclear (*n* = 1)

AVS, acute vestibular syndrome; CVS, chronic vestibular syndrome; EVS, episodic vestibular syndrome; PPPD, persistent postural-perceptual dizziness.

Four patients with BRV showed canal paresis on the caloric test, one out of these four patients had a unilateral gain reduction of horizontal and anterior canals, and one patient had a gain reduction of the posterior canal during the vHIT test.

## Discussion

In the present study, we found that central vestibular disorders should be highly suspected when patients showed pure UBN or SN with a predominant upbeat component. For vestibular disorders presenting as SN with an upbeat component, PCI and VM were seen more commonly in patients with central vestibular disorders, whereas AUVP was the most common in peripheral vestibular disorders.

### AUVP

We found that most patients with AVS who showed SN with an upbeat component had AUVP (mainly superior AUVP), and these patients were accompanied by horizontal and anterior semicircular canals hypofunction. The types of SN in these patients were mixed UBN-HBN with or without a torsional component, and the upbeat component of the nystagmus was significantly weaker than the horizontal component. The generation of the upbeat component is mainly associated with hypofunction in the anterior semicircular canal afferents, which would result in relative excitation of the opposite posterior semicircular canal, thus producing UBN with or without a torsional component, whereas the horizontal component was associated with hypofunction in the horizontal semicircular canal afferents.

Previous studies investigated the presence of spontaneous vertical-horizontal-torsional nystagmus in patients with AUVP and found that the vertical component of nystagmus was mostly UBN, and the intensity of the upbeat component was weaker than the horizontal component (14, 16, 29). This may be related to the fact that AUVP/VN primarily involves the superior branches, mainly including the anterior and horizontal semicircular canals, and the utricle (30), because there are anatomical differences between the superior vestibular nerve (SVN) and the inferior vestibular nerve (IVN). The bony channel of the SVN is seven times longer than that of the IVN, and the SVN channel has a larger percentage of bony spicules than the IVN channel, making the SVN more susceptible to entrapment and ischemia (30).

### PCI

We found that PCI is the second most common cause of spontaneous UBN in patients with AVS. In patients with PCI, the lesions occur mainly in the medial part of the medulla oblongata, pons, or midbrain, and infarction over the ventral part of the pons, lateral part of the medulla or cerebellum can also be seen. The types of nystagmus in these patients were mixed nystagmus, including UBN-HBN-TN, UBN-HBN, and UBN-TN. The upbeat component of the SN was stronger than the horizontal component in 55.6% (5/9) of patients with PCI, which was weaker than the horizontal component in four patients [including lateral medullary infarction (*n* = 1), medial medullary infarction (*n* = 1), medial pontine infarction (*n* = 1), and infarction involving lateral medulla and cerebellum (*n* = 1)]. A previous study has shown that the presence of SN with an upbeat component in medial medullary infarction is mainly associated with the involvement of the perihypoglossal nucleus (including the nucleus prepositus hypoglossi (31, 32), nucleus intercalatus and nucleus of Roller (19) or damage to the vestibulo-ocular reflex (VOR) pathway of anterior semicircular canal (33). In contrast, the occurrence of SN with an upbeat component in lateral medullary infarction is associated with the involvement of vestibular nuclei, because damage to the vestibular nuclei can lead to the occurrence of various types of SN (2). Similarly, for patients with pontine tegmentum infarction, the presence of upbeat nystagmus is mostly due to the damage of the ascending VOR pathway from the anterior semicircular canal to ocular motor nuclei *via* the medial longitudinal fasciculus (2). For focal infarct occurs between the base and the tegmentum of the pons at its middle-to-upper level, the presence of UBN may be due to damage to the ventral tegmental tract, which is also one of the ascending VOR pathways (34).

### Possible labyrinthine infarction and isolated acute unilateral utricular vestibulopathy

In this study, SN with an upbeat component was often seen in other rare peripheral vestibular disorders, including possible labyrinthine infarction and isolated acute unilateral utricular vestibulopathy. Studies have shown that inner ear ischemia may cause dizziness/vertigo since the inner ear requires high energy metabolism and lacks collateral circulation (35, 36). Because the internal auditory artery is a terminal artery with insufficient collateral circulation, the vestibular labyrinth and its various components appear more vulnerable to ischemia (37). Therefore, internal auditory artery infarction can often lead to severe peripheral vestibular disorders and hearing loss (37). However, it is currently difficult to visually confirm the presence of labyrinthine infarction by imaging (38). One patient in the present study had horizontal and anterior canal hypofunction, ipsilateral hearing loss, risk factors for atherosclerosis, and a normal MRI. We suggest that the patient may have a labyrinthine infarction. In one patient with isolated acute unilateral utricular vestibulopathy included in the present study, the types of SN were UBN-HBN, oVEMP test results on the right side were abnormal, whereas vHIT, caloric test, gaze, saccade, smooth pursuit, and optokinetic reflex test results, and brain MRI findings were normal in the patient. Isolated acute unilateral utricular vestibulopathy has rarely been studied (39). A previous case report of a patient with unilateral utricular dysfunction showed that the patient had SN with horizontal and vertical components during an acute attack phase. vHIT findings showed normal VOR gain in both the horizontal and vertical semicircular canals. It is speculated that the neural input from the otoliths may converge on horizontal semicircular canal neurons in the vestibular nuclei. When the utricular function is asymmetrical, SN, like the nystagmus caused by asymmetrical semicircular canal function, is produced (39). The mechanism underlying the occurrence of SN in isolated acute unilateral utricular vestibulopathy remains to be further investigated.

### BRV

In recent years, clinicians have found a group of recurrent vertigo syndromes presenting as spontaneous vertigo or instability, which is usually not accompanied by hearing loss, tinnitus, or aural fullness, as well as migraine or migraine aura during the acute phase (28). The etiologies are not the recognized episodic vestibular syndromes, such as VM, MD, benign paroxysmal positional vertigo, and vestibular paroxysmia (40). A previous study has shown that patients with BRV do not have unique clinical features compared to those with VM and MD (41). During the follow-up period, the diagnosis of BRV was changed to VM or MD in a small proportion of patients (42, 43). A previous study documented no statistically significant differences in the frequency of vertigo attacks between patients with BRV and patients with MD and VM after 6 months and 3 years of follow-up (40). MRI revealed endolymphatic hydrops in about 23% of patients with BRV, which was more frequently involved in the horizontal semicircular canal (59%), and patients with BRV often showed abnormal caloric test results (49%) (28). Attyé et al. (44) found that 48.4% of patients with BRV showed vestibular hydrops and/or cochlear hydrops on MRI. Some authors suggest that there is a subtype of vestibular MD presenting with recurrent attacks of vertigo without fluctuating hearing loss and symptoms of aural fullness (45). In the present study, BRV was the most common cause of spontaneous UBN in patients with EVS, two of the four patients with BRV showed mixed SN with a non-predominant upbeat component, and the other two showed pure UBN. Even though these patients showed evidence of lesions involving the peripheral vestibular system on the caloric test or vHIT test, we cannot wholly exclude the central origin, especially in patients with pure UBN.

### VM and VM of childhood

In this study, three patients with VM showed SN with an upbeat component. Of these three patients, two patients had purely UBN, and one patient had UBN-HBN. Patients with VM may show spontaneous or positional vertigo, and some patients may experience a change from spontaneous vertigo to positional vertigo after a few hours or days (25, 46). Studies have shown that the incidence of ictal SN in VM patients ranged from 9.9 to 71.3% (47–50), which was mostly HBN (47, 48, 50). SN in patients with VM suggests an imbalance in the peripheral or central vestibular system (51). One study described the clinical characteristics of 19 patients with VM and found that one out of these 19 patients developed spontaneous UBN, which resolved after treatment with external trigeminal nerve stimulation (52). Another study showed that the incidence of spontaneous vertical nystagmus in the absence of fixation was 16%. Unfortunately, the authors did not describe the proportion of patients presenting vertical nystagmus with an upbeat component (49). It has been shown that some patients with VM have AUVP, mostly showed unilateral canal paresis of the horizontal semicircular canal during the caloric test and normal vHIT results (50). In the present study, one patient with VM had canal paresis of the horizontal semicircular canal, however, vHIT showed normal VOR gains of the horizontal and vertical semicircular canal.

In the present study, we also found the presence of spontaneous UBN in two children with VM. A population-based study showed that the prevalence of recurrent vertigo that may be associated with migraine in children aged 6–12 years was estimated to be 2.8% (53). Similar to VM in adults, SN, positional nystagmus, or head-shaking nystagmus can also be seen in pediatric patients with VM (54). The rate of spontaneous UBN detected during funduscopy in children with definite VM has been reported to be 6% (54). Marcelli et al. (55) found that the prevalence of positional nystagmus was 44%, while the prevalence of head-shaking nystagmus was 31% in children with migraine and vestibular symptoms.

### Other rare types of episodic vestibular disorders

In this study, one patient with probable MD showed mixed spontaneous UBN-HBN with the horizontal component toward the healthy side, the caloric test showed canal paresis on the left side, but vHIT showed normal gains in all semicircular canals. A previous study showed that 42–77% of patients with unilateral MD had canal paresis on the caloric test (56), and semicircular canal VOR gains were mostly normal during the high-frequency head impulse test (57). Therefore, the presence of mixed spontaneous UBN-HBN in this patient with probable MD may be associated with simultaneous impairment of the horizontal and anterior canal function. In addition, in the present study, one patient was found to have postoperative fourth ventricle ependymoma. Previous studies have reported the presence of spontaneous or positional UBN in patients with occupying lesions within the fourth ventricle (58, 59). The presence of spontaneous UBN may be related to fourth ventricular occupying lesions or surgical damage to the adjacent brainstem or cerebellar structures. Additionally, in the present study, we also found UBN in patients with brainstem encephalitis, autoimmune encephalitis, and TIA. Similar findings have also been reported in previous studies (3, 60, 61).

### Wernicke's encephalopathy

In the present study, a patient with Wernicke's encephalopathy was observed. Axial FLAIR sequence showed hypersignals in the midbrain periaqueductal gray matter. The patient showed purely UBN, which disappeared after 8 months of follow-up. Wernicke's encephalopathy is caused by thiamine (vitamin B1) deficiency, and patients with Wernicke's encephalopathy may present with spontaneous vertical nystagmus (62). It has been suggested that the presence of spontaneous vertical nystagmus in patients with Wernicke's encephalopathy may be caused not only by imbalances in the semicircular canal-ocular reflex pathways governing the rotational VOR, but also by an imbalance in the otolith-ocular pathways that govern the linear VOR (18). Studies have shown that in the acute phase of Wernicke's encephalopathy, patients presented with spontaneous UBN in primary gaze, which may change to permanent DBN in the chronic phase of Wernicke's encephalopathy (18, 63). Spontaneous UBN may obviously diminish or change to DBN with a change in convergence and horizontal or vertical gaze, head shaking, or mastoid vibration (18, 63). However, in the present study, the conversion of UBN to DBN was not found during the follow-up period. A study analyzed the changes in the characteristics of positional nystagmus in 13 patients with Wernicke's encephalopathy and found a conversion of UBN to permanent DBN in seven patients during the follow-up period. The underlying mechanisms for the occurrence of direction-reversing nystagmus in patients with Wernicke's encephalopathy may be the directional vulnerability of the vertical gaze-holding networks in the dorsomedial medulla to thiamine deficiency and the impairment in the processing of otolithic information (18, 63, 64).

### Limitations

Our study has two main limitations. First, the study was conducted at a single-center tertiary hospital run primarily by neurologists. Therefore, selection bias is unavoidable in the inclusion of cases, and the results cannot be generalized to other primary clinics or clinics mostly run by otolaryngology department doctors. Second, we could not quantify the torsional component of nystagmus due to using 2D video-oculography. When the torsional component of the nystagmus is minimal, the examiner may misjudge the nystagmus as pure UBN due to the limited ability of visual recognition of the torsional component.

## Conclusions

Our findings suggested that central vestibular disorders should be highly suspected when pure UBN or SN with a predominant upbeat component was observed. In vestibular disorders presenting as SN with an upbeat component, PCI and VM were the most frequently seen among patients with central vestibular disorders, whereas AUVP was the most common in peripheral vestibular disorders.

## Data availability statement

The raw data supporting the conclusions of this article will be made available by the authors, without undue reservation.

## Ethics statement

The studies involving human participants were reviewed and approved by IRB No 2022(023). The patients/participants provided their written informed consent to participate in this study.

## Author contributions

XL and Y-XW acquired and analyzed the data and wrote the manuscript. Y-FF and T-TZ collected the data and interpreted the results. G-PZ and J-SK interpreted the results and revised the manuscript. Z-XW and XY conceptualized the study, interpreted the results, and revised the manuscript. All authors contributed to the article and approved the submitted version.
